# Secondary Subacromial Impingement after Valgus Closing-Wedge Osteotomy for Proximal Humerus Varus

**DOI:** 10.1155/2015/652096

**Published:** 2015-04-27

**Authors:** Hirotaka Sano, Masayuki Kamimura, Akira Oizumi, Shuji Isefuku

**Affiliations:** ^1^Division of Orthopedics, Sendai City Hospital, 1-1-1 Asutonagamachi, Taihaku-ku, Sendai 982-8502, Japan; ^2^Department of Orthopaedic Surgery, Tohoku University School of Medicine, Sendai, Japan; ^3^Department of Orthopaedic Surgery, Kurihara Central Hospital, Miyagi, Japan; ^4^Department of Orthopaedic Surgery, Sendai Medical Center, Sendai, Japan

## Abstract

A 31-year-old construction worker had been suffering from both the motion pain and the restriction of elevation in his right shoulder due to severe varus deformity of humeral neck, which occurred after proximal humeral fracture. The angle for shoulder flexion and abduction was restricted to 50 and 80 degrees, respectively. Valgus closing-wedge osteotomy followed by the internal fixation using a locking plate was carried out at 12 months after injury. Postoperatively, the head-shaft angle of the humerus improved from 65 to 138 degrees. Active flexion and abduction angles improved from 80 to 135 degrees and from 50 to 135 degrees, respectively. However, the patient complained from a sharp pain with a clicking sound during shoulder abduction even after removal of the locking plate. Since subacromial steroid injection temporarily relieved his shoulder pain, we assumed that the secondary subacromial impingement was provoked after osteotomy. Thus, arthroscopic subacromial decompression was carried out at 27 months after the initial operation, which finally relieved his symptoms. In the valgus closing-wedge osteotomy, surgeons should pay attention to the condition of subacromial space to avoid causing the secondary subacromial impingement.

## 1. Introduction

Severe varus deformity of the humeral neck has been reported as a relatively rare complication after proximal humeral fracture in younger population [1–4], which causes motion pain as well as restriction of range of motion in the shoulder joint. Previous authors reported that valgus osteotomy at the humeral neck provided a marked improvement of these symptoms [1–4]. However, the potential pitfalls of this procedure have not yet been fully clarified, since the number of patients was limited. The patient reported in this paper demonstrated secondary subacromial impingement after successful valgus closing-wedge osteotomy, which required another surgery for subacromial decompression.

## 2. Case Presentation

A 31-year-old construction worker was injured by the traffic accident and was carried to the emergency care unit in the authors' institution. The primary diagnoses were multiple fractures (facial bones, ribs, right humeral surgical neck, and pelvic bones), hemothorax, and urethral rupture. He underwent an emergent operation for the treatment of hemorrhagic shock due to multiple thoracoabdominal traumas. He had been kept in the intensive care unit for one month. After improvement of his general condition, he was referred to the authors' outpatient clinic for the treatment of right proximal humeral fracture. Since the patient was reluctant to undergo another surgery at that time, he was treated conservatively. However, neither the pain nor the range of motion improved despite of 6-month intensive rehabilitation. The range of motion for shoulder flexion and abduction was restricted to 50 and 80 degrees, respectively. On the other hand, no severe restrictions were seen in other directions; that is, extension: 45 degrees, external rotation: 50 degrees, and internal rotation: L1 level.

**Figure 1:**
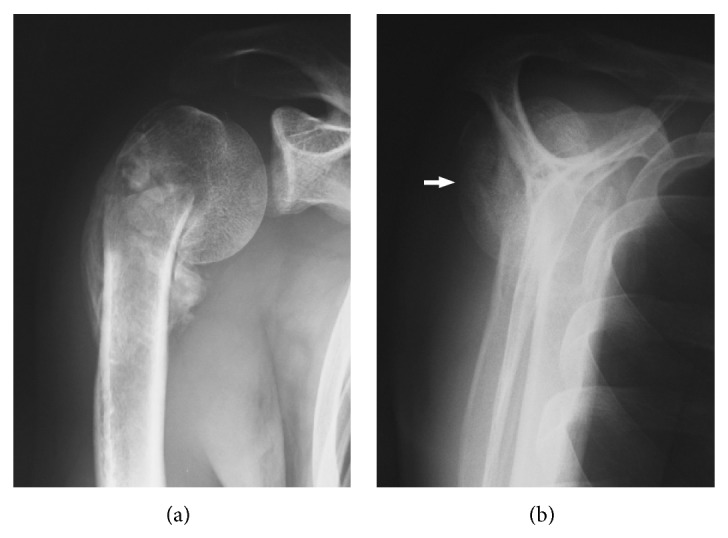
Preoperative anteroposterior (a) and scapula-Y (b) radiographs demonstrate varus and retroversion (arrow) deformity of the humeral neck.

**Figure 2:**
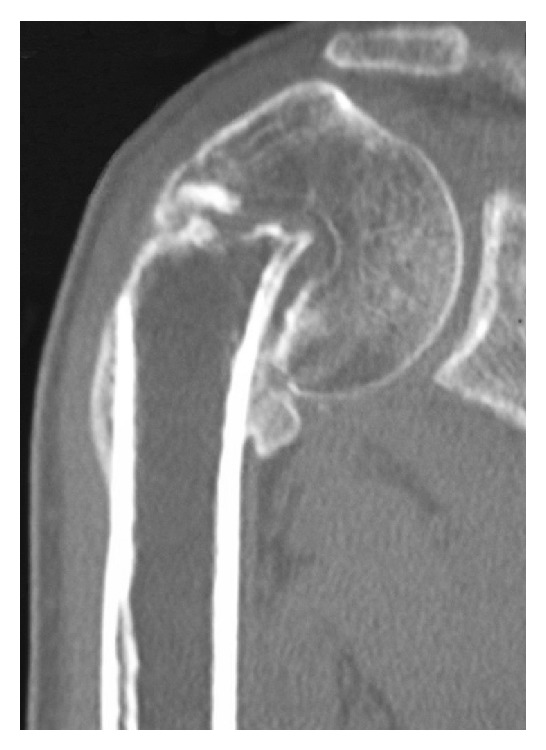
Preoperative CT image (oblique coronal plane) clearly shows that the greater tuberosity locates just beneath the undersurface of acromion.

**Figure 3:**
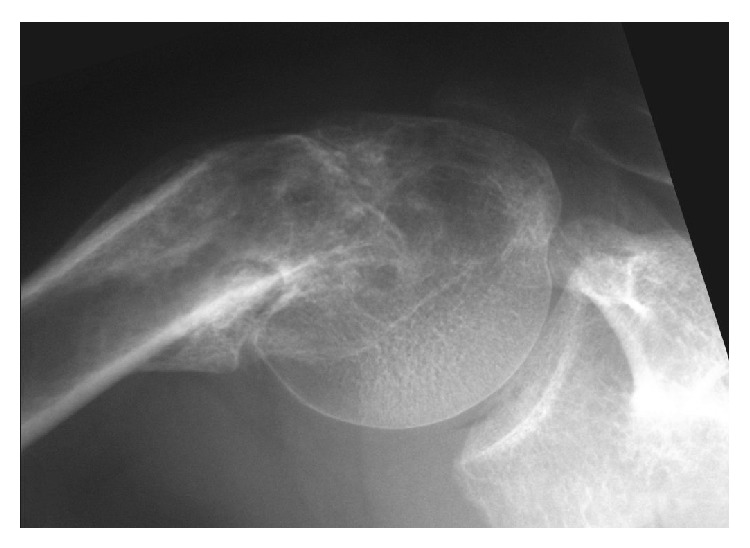
In fluoroscopic dynamic observation, greater tuberosity smoothly passes under the acromion without causing impingement; however, it does come into contact with the upper glenoid rim, which seems to block further glenohumeral abduction.

**Figure 4:**
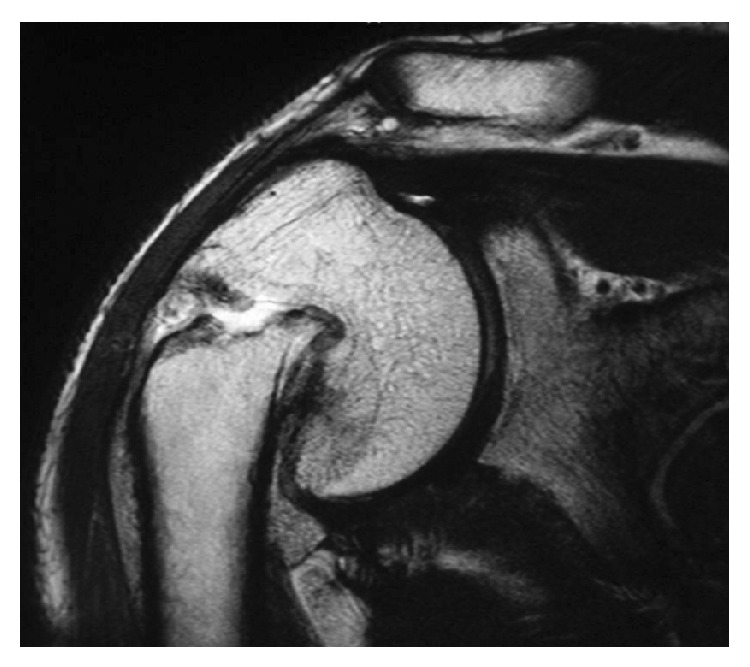
Preoperative T2-weighted MR image shows neither the rotator cuff tear nor the fluid collection in the subacromial bursa.

Radiologically, a marked varus deformity with an excessive retroversion was seen in the proximal humerus, which was caused by a malunited surgical neck fracture (Figures 1(a) and 1(b)). The neck-shaft angle measured with the anteroposterior radiograph [2, 5] was 65 degrees. Computed tomographic (CT) images clearly demonstrated the extent of varus deformity. Although the greater tuberosity located just beneath the acromion at the hanging arm position, no spur formation was seen on the undersurface of the acromion (Figure 2). Fluoroscopic observation revealed that the greater tuberosity did not impinge against the acromion during shoulder abduction. Instead, it came into contact with the upper glenoid rim, which seemed to block further shoulder abduction (Figure 3). No tears were seen in rotator cuff tendons in T2 weighted MR images (Figure 4).

**Figure 5:**
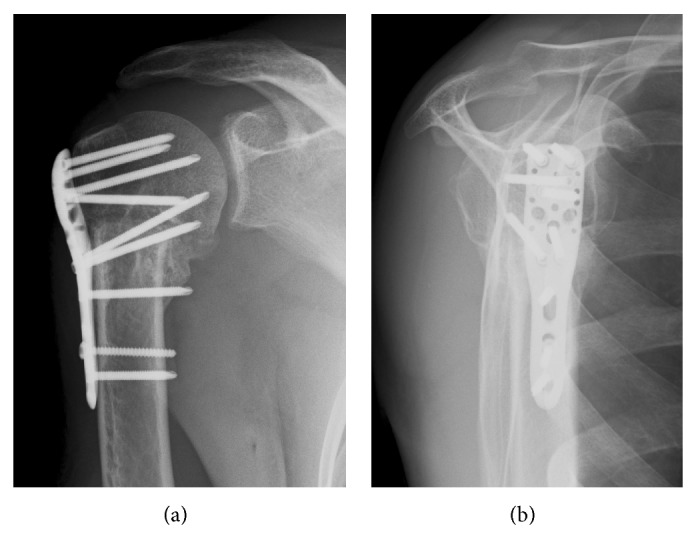
Both varus and retroversion deformities are successfully corrected after valgus closing-wedge osteotomy ((a) anteroposterior view, (b) scapula-Y view).

Based on these findings, valgus closing-wedge osteotomy was performed at 12 months after the injury. In the surgical procedure, proximal part of humerus was exposed via deltopectoral approach. No tear was found on the bursal surface of rotator cuff tendons. The angle of wedge osteotomy was determined as 50 degrees based on the preoperative radiological measurement. The rotational deformity was corrected by aligning bicipital groove as a landmark. After checking the alignment of the osteotomy site using fluoroscopy, two fragments were fixed with a PHILOS locking plate (Synthes, USA). The head-shaft angle improved to 138 degrees and the position of greater tuberosity became lower than that of the superior margin of humeral head. Rotational deformity was also properly corrected (Figures 5(a) and 5(b)). Postoperatively, the operated arm was immobilized with an arm sling for three weeks and passive mobilization as well as the pendulum exercise was performed during this period. Then, the sling was removed and active exercises were permitted.

**Figure 6:**
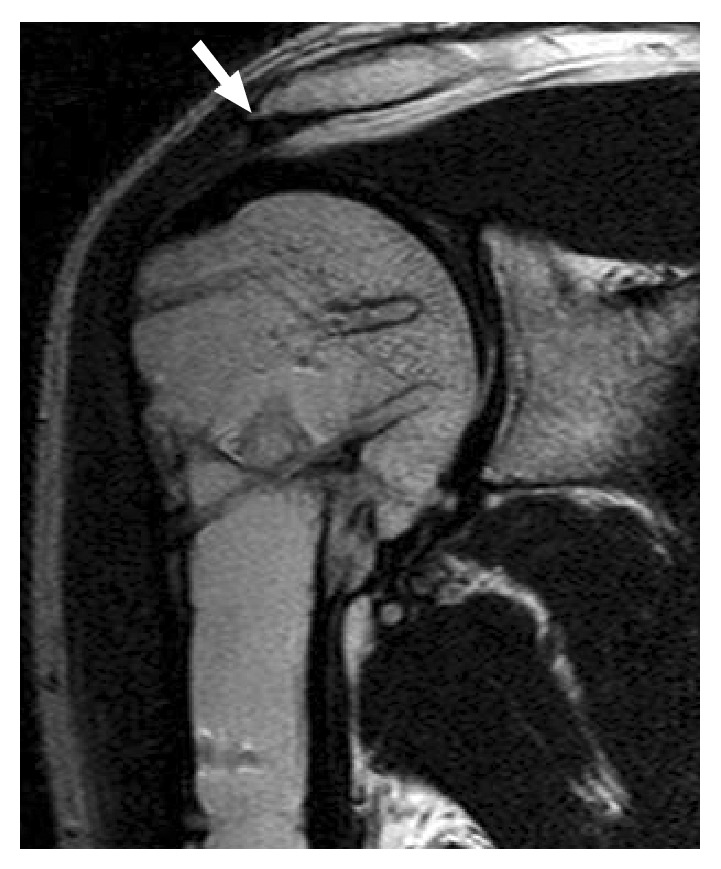
Although no fluid collection is seen in the subacromial bursa, the presence of a newly formed spur formation (arrow) is confirmed at the lateral margin of acromion (oblique coronal T2-weighted MR image).

At 16 months after the surgery, union of the osteotomy site was completed and both flexion and abduction angles improved to 135 degrees. However, he complained a sharp motion pain with a clicking sound around 90-degree abduction. Subacromial steroid injection relieved his shoulder pain immediately but it lasted only for a few days. The plate was removed via deltopectoral approach at 20 months after the osteotomy since we assumed it was responsible for the subacromial impingement. To our disappointment, however, it did not contribute to relieve his shoulder pain at all. T2-weighted MR images taken at this time demonstrated the presence of a small subacromial spur although no fluid collection was seen in the subacromial bursa (Figure 6). Thus, another surgery for arthroscopic subacromial decompression was performed at 27 months after the osteotomy. The presence of thickened bursal tissue was confirmed in the subacromial space, which was debrided using a shaver. A small bony spur was also removed from the undersurface of the acromion. After this operation, both the motion pain and the clicking sound subsided in his right shoulder. Although the patient still had a slight pain during shoulder abduction, he had successfully returned to his original job. Both active flexion and abduction angles reached 140 degrees at 36 months after the osteotomy.

## 3. Discussion

In the varus deformity of the proximal humerus, previous authors believed that proximity of the greater tuberosity caused a subacromial impingement, which was responsible not only for the shoulder pain [2, 4] but also for the restriction of elevation [3, 4]. However, Benegas et al. described that the restriction of elevation was mainly caused by the reduction of both lever arm of supraspinatus muscle and sliding surface between humeral head and glenoid [2]. The patient presented in this paper showed no impingement preoperatively between the greater tuberosity and the acromion during shoulder abduction. Instead, fluoroscopic observation revealed that abduction of the glenohumeral joint was blocked due to the contact of the greater tuberosity to the upper glenoid rim. Soft tissue pathologies including shortening of supraspinatus muscle as well as global adhesion of pericapsular tissue may also contribute to development of the restriction of range of motion. To solve all these problems, we determined to perform the valgus closing wedge-osteotomy for this patient.

Radiologically, the alignment of the proximal humerus was successfully corrected with the osteotomy. The head-shaft angle improved to 138 degrees and position of greater tuberosity became lower than that of the superior margin of humeral head, which seemed to be acceptable [4]. Nevertheless, secondary subacromial impingement was provoked postoperatively. Since the removal of locking plate did not contribute to the pain relief, arthroscopic subacromial decompression was performed. Such clinical course in this patient suggested that secondary subacromial impingement could be provoked after valgus closing-wedge osteotomy even if the bony alignment seemed to be acceptable radiologically. We believed that surgeons should carefully check the condition of subacromial space when performing this procedure. Moreover, they should not hesitate about readjusting the alignment of the osteotomy site to avoid causing secondary impingement, if there was a narrowing of the subacromial space.

## 4. Conclusions

Valgus closing-wedge osteotomy provided a marked improvement of the bony alignment as well as the range of motion in a patient with severe varus deformity of the proximal humerus. However, care should be taken for the condition of subacromial space during this procedure to avoid secondary subacromial impingement.
